# IDTI Dyes for Fluoride Anion Chemosensors

**DOI:** 10.3389/fchem.2020.591860

**Published:** 2020-10-23

**Authors:** Xinqiang Yuan, Xin Shi, Cheng Wang, Yuqian Du, Peng Jiang, Xizhou Jiang, Yongqiang Sui, Xiaoli Hao, Lin Li

**Affiliations:** ^1^National and Local Joint Engineering Laboratory for Slag Comprehensive Utilization and Environmental Technology, School of Materials Science and Engineering, Shaanxi University of Technology, Hanzhong, China; ^2^Key Laboratory of Rubber-Plastics of Ministry of Education/Shandong Province, School of Polymer Science & Engineering, Qingdao University of Science & Technology, Qingdao, China; ^3^Prinx Chengshan (Shan Dong) Tire Co., Ltd., Rongcheng, China

**Keywords:** IDTI, chemosensors, fluoride anion, color changing, spectroscopy

## Abstract

Fluoride anions play a key role in human health and chemical engineering, such as in organic synthesis and biological processes. The development of high-sensitivity naked-eye detection sensors for fluoride anions in organic solutions is crucial and challenging. In this study, (3Z,3′Z)-3,3′-[4,4,9,9-tetrakis(4-hexylphenyl)-4,9-dihydro-s-indaceno(1,2-b:5,6-b′)dithiophene]-2,7-diylbis(methan-1-yl-1-ylidene) bis(6-bromo-indolin-2-one) (IDTI) was designed and used as a fluoride chemosensor for the first time. IDTI is a highly sensitive fluoride sensor with a detection limit of as low as 1 × 10^−7^ M. In addition, upon the reaction of IDTI with fluoride anions in a tetrahydrofuran (THF) solution, color changes from red to yellow under ambient light and from purple to green under UV light were detectable by the naked eye. These studies indicate that IDTI is a promising fluoride chemosensor.

**Graphical Abstract:**
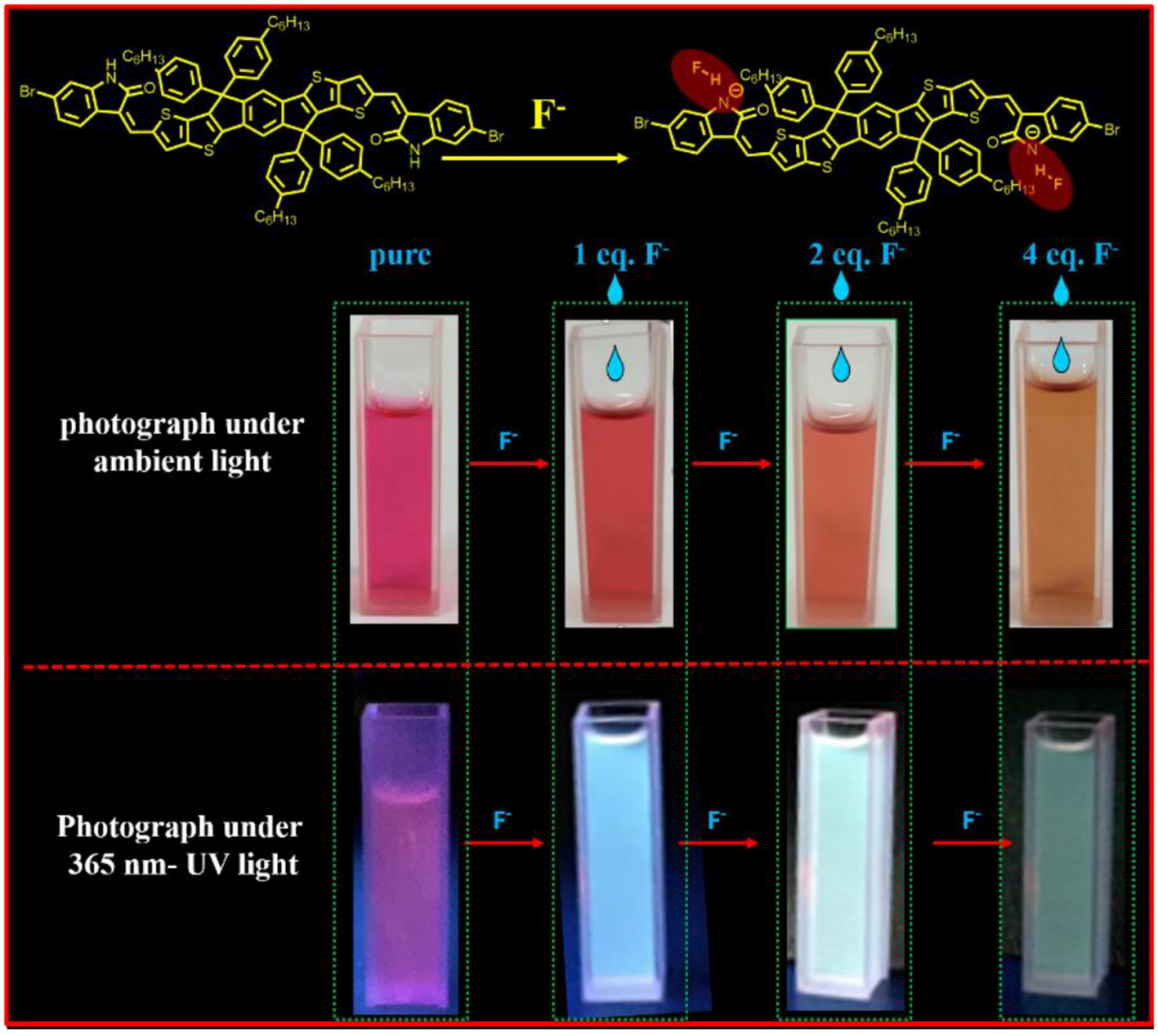
The new dye, IDTI, firslty used as a fluoride anion chemosensor, has a high sensitivity with the fluoride anion detection limit of as low as 1 × 10^−7^ M. And the IDTI solutions have the interesting color changing properties, once meet the fluoride anion, from red to yellow under ambient light and from purple to green under UV light were detectable by the naked eye. The IDTI is a promising fluoride chemosensor.

## Introduction

The development of high-efficiency and sensitive fluoride anion (F^−^) sensors is crucial, since F^−^ plays a key role in chemical military fields, industry, organic synthesis, biological and medical processes, and even human health (Wade et al., 2011; Li et al., 2012; Yang et al., 2012, 2013; Sun et al., 2017). With the rapid development of the chemical industry, F^−^ exists not only in aqueous environments but also in organic media, such as waste organic liquor. As such, several different optical chemosensors for fluoride anions in organic solutions have been developed (Zhang et al., 2018). However, the most reported fluoride anion sensors can only detect F^−^ at concentrations in the 10^−5^ M level.

Fluoride anions, which are among the smallest anions, exhibit strong electronegative properties. Such properties enable F^−^ to form strong hydrogen bonds with –NH groups or easily deprotonate the –NH protons of the designed receptor, resulting in an evident optical spectrum change either in the absorption or emission spectrum (Kaur and Choi, 2015; Feng et al., 2018). Some sensors can even change their color under ambient light or UV light, with the change detectable by the naked eye (Wang et al., 2018; Antonio et al., 2020). Recently, Zhang et al. (2018) reported polymers containing 1,4-diketo-pyrrolo[3,4-c]pyrrole (DPP)-substituted *t*-butoxy carbonyl (*t*-Boc) units with good solubility. After thermal annealing processing, the *t*-Boc units could be removed, and lactam hydrogen appeared. They reported that the resulting polymer cannot only be used for the detection of F^−^ but also the extraction of F^−^ from the organic solution. Later, Wu et al. (2019) designed a dye containing hydroxyl to detect F^−^ in dimethyl sulfoxide (DMSO); this approach exhibited a detection limit of as low as 1.79 μM. Deng et al. (2020) reported aminobenzodifuranone dyes for F^−^ chemosensors, which could not only detect F^−^ but also distinguish solvents containing F^−^. Hitherto, large optical spectrum shifts, obvious color changes that can be detected by the naked eye, and sensitive fluoride chemosensors are still rare and quite important. In this work, a new π-conjugated dye, (3Z,3′Z)-3,3′-[4,4,9,9-tetrakis(4-hexylphenyl)-4,9-dihydro-s-indaceno(1,2-b:5,6-b′)dithiophene]-2,7-diylbis(methan-1-yl-1-ylidene)bis(6-bromo-indolin-2-one) (IDTI), was designed and used as a fluoride anion sensor for the first time. This dye exhibited promising sensitivity toward F^−^ in organic solvents.

## Materials and Methods

The procedure for the synthesis of IDTI is as follows. In a 50-ml round-bottomed flask, 6-bromooxindole (0.63 g, 3.0 mmol) and 2,7-dicarbaldehyde-4,4,9,9-tetrakis(4-hexylphenyl)-4,9-dihydro-s-indaceno(1,2-b:5,6-b′)dithiophene **1** (1.074 g, 1.0 mmol) were suspended in absolute dry alcohol (15 ml). Subsequently, piperidine (0.25 ml) was added using a syringe needle. The reaction mixture was then stirred and refluxed for 18 h. After cooling to room temperature, the mixture was filtered, washed with ethanol, and dried under vacuum to yield a brown powder product (1.23 g, 84.2%). Microanalysis found C, 70.47%; H, 5.68%; N, 1.96%; S, 8.80% (C, 70.57%; H, 5.65%; N, 1.91%; S, 8.76%).

## Results and Discussion

**Figure 1:**
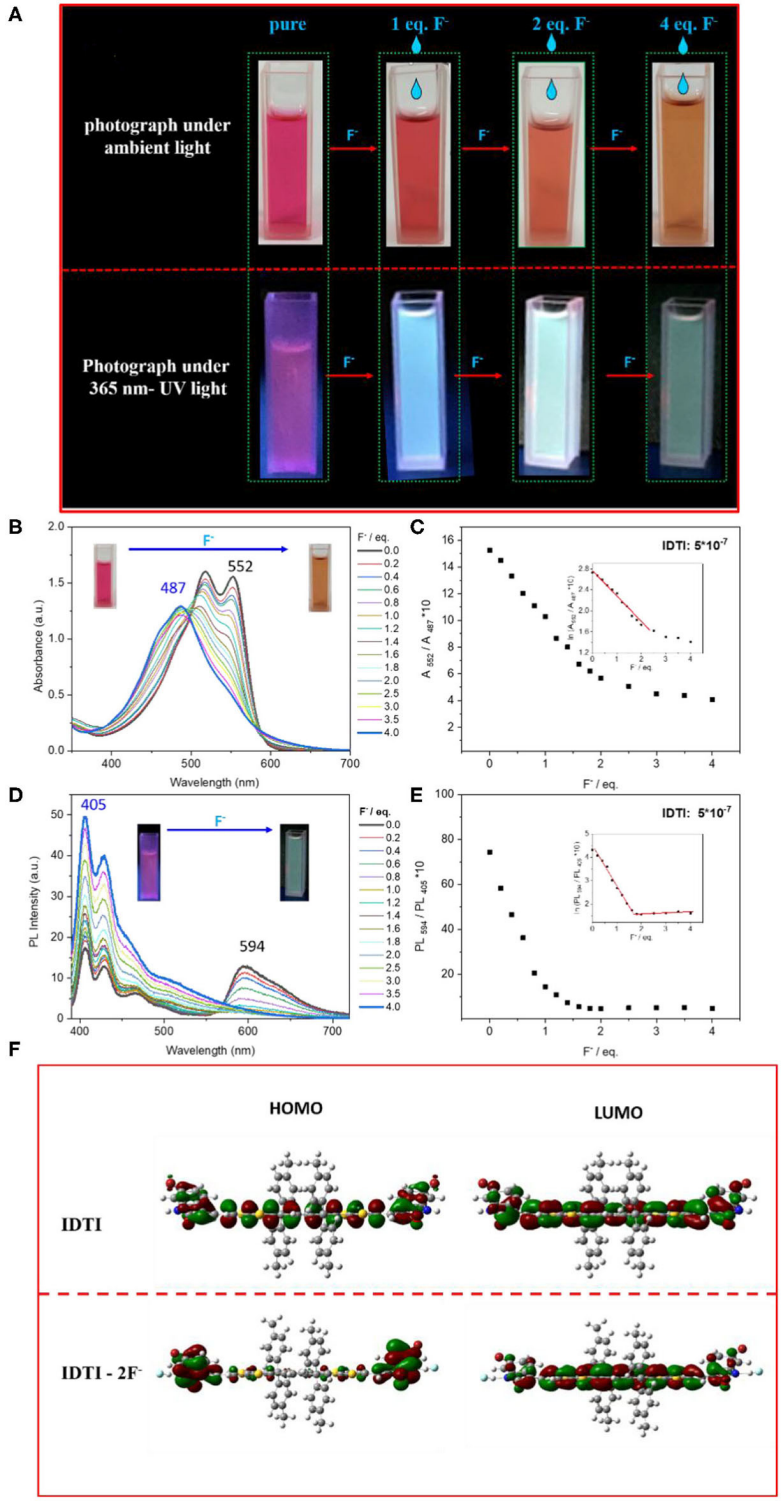
**(A)** Photograph of the IDTI solution with or without additional different eq. fluoride anion under ambient light or 365 nm UV light. **(B,D)** UV/Vis absorption **(B)** and PL fluorescence spectra **(D)** of IDTI (5.0 × 10^−7^ M) in the presence of F^−^ (0–4.0 eq.) in tetrahydrofuran (THF). **(C,E)** Ten times the absorption intensity ratio of IDTI (5 × 10^−6^ M after mixing with F^−^ in THF) between 552 and 487 nm (A_552_/A_487_ nm) vs. fluoride anion concentration **(C)** and the PL fluorescence intensity ratio of IDTI (5 × 10^−6^ M after mixing with F^−^ in THF) between 594 and 405 nm (PL_594_/PL_405_ nm) **(E)** vs. fluoride anion concentration. The inset is the natural logarithm of 10 times the absorption intensity ratio A_552_/A_487_ nm and PL_594_ /PL_405_ nm vs. fluoride anion concentration, respectively. **(F)** Molecular orbital surfaces of the highest occupied molecular orbital (HOMO) and lowest unoccupied molecular orbital (LUMO) energy levels of IDTI and IDTI-2F^−^ obtained at the B3LYP/6-31G* level.

**Scheme 1:**
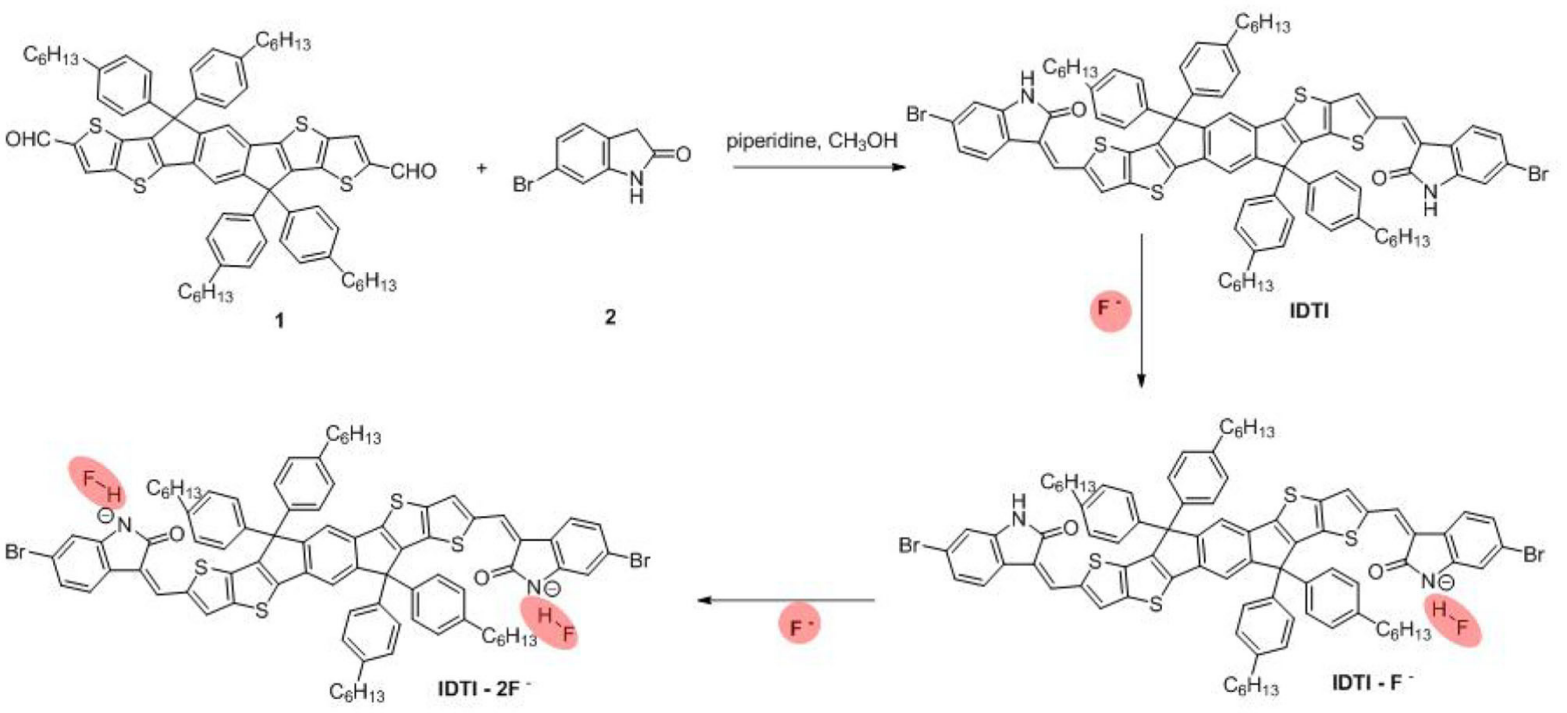
Synthetic route for IDTI and the intermolecular proton transfer between –NH units from IDTI and F^−^.

The produced IDTI exhibited low solubility in most common organic solvents; however, such solubility was sufficient for studying the behavior of the fluoride anion chemosensor. The interaction between the IDTI chromophore and F^−^ was first investigated through color changes that were detectable by the naked eye. Tetrabutylammonium fluoride (TBAF, 5.0 × 10^−4^ M) was gradually added to a 1.0 × 10^−5^ M solution of IDTI in tetrahydrofuran (THF). As shown in Figure 1A, the pure IDTI in THF exhibited a rose color under ambient light and a purple color under UV light (365 nm). However, once F^−^ was added, color changes were observed under both light sources. With the addition of 1, 2, and 4 eq. of F^−^, the color of the IDTI solution under irradiation of ambient light changed from rose to red, orange, and yellow, respectively. Likewise, the emission color gradually changed from purple to blue, cyan, and green with the corresponding amounts of F^−^. This result indicates that IDTI could be used to detect F^−^ through color changes that can be observed with the naked eye. Such color changes could be due to the deprotonation of the lactam NH moiety of the IDTI core by F^−^ [intermolecular proton transfer (IPT), Scheme 1] (Chen et al., 2012, 2017). IDTI exhibited electronically neutral properties in the absence of F^−^. However, once F^−^ was added, F^−^ could react with the NH units of the IDTI core to form IDTI-F^−^; the IPT process resulted in a color change of the dye. Furthermore, with higher concentrations of F^−^ in the solvent, further IPT could proceed between F^−^ and NH to produce IDTI-2F^−^ (Sun et al., 2017).

The above color changes were further characterized by spectroscopy. Instead of an IDTI concentration of 1.0 × 10^−5^ M, 5.0 × 10^−7^ M IDTI in THF was investigated. As shown in Figure 1B, the spectrum for pure IDTI in the THF solution exhibited a maximum absorption at 552 nm and a shoulder peak at 515 nm. With the gradual addition of F^−^, the two peaks located at 552 and 515 nm successively decreased and became blue shifted, which finally disappeared. Moreover, a new peak at 487 appeared at 1.4 eq. of ammonium fluoride and became progressively higher with increasing F^−^ concentration. These absorption spectra showed a clear isosbestic point at 495 nm. To examine the sensitivity of IDTI toward F^−^, we investigated the radiometric curves. Figure 1C shows the graph of 10 times the absorption intensity ratio of IDTI between 552 and 487 nm vs. the F^−^ concentration, which appears to be a quadratic function; the inset shows the graph of the natural logarithm of these data. The inset of Figure 1C shows one slope in the correlation, and the linear relationship between the absorption intensity and F^−^ concentration can be described by the equation: ln(A_552_/A_487_ nm) = −0.50 [F^−^] + 2.76 ([F^−^] <1.0 × 10^−6^ M). Figure 1D shows the fluorescence spectra of IDTI (5.0 × 10^−7^ M) in the presence of F^−^ (0–4.0 eq.) in THF. The fluorescence spectra exhibited the same trend as the absorption spectra for IDTI; in particular, a blue shift of the spectra was observed upon the addition of F^−^ to the THF solvent. Pure IDTI in the THF solution exhibited maximum emission at 594 nm. With the addition of F^−^, this peak successively decreased, while the peak at 405 nm progressively increased. Figure 1E shows the graph of 10 times the fluorescence intensity ratio of IDTI between 594 and 405 nm vs. the F^−^ concentration, while the inset shows the graph of the natural logarithm of these data. The inset of Figure 1E shows the presence of two slopes in the correlation, and the linear relationship between the absorption intensity and F^−^ concentration at low concentrations can be described by the following equation: ln (A_594_/A_405_ nm) = −0.71 [F^−^] + 4.3 ([F^−^] <8 × 10^−7^ M). As shown in Figures 1C,E, both the absorption spectra and PL fluorescence spectra showed a clear distinction between the solution with an F^−^ concentration of 1.0 × 10^−7^ M and the pure IDTI solution, which indicates that IDTI is a sensitive fluoride chemosensor. The linear relationship between the absorption/PL fluorescence intensity and F^−^ concentration indicates that IDTI could be used to quantitatively analyze the fluoride concentration.

To understand the electron distributions before and after F^−^ binding, the frontier molecular orbital (FMO) feature was calculated at the B3LYP/6-31 (d, p) level using simplified IDTI, in which the long hexyl chain was replaced with a methyl group. As shown in Figure 1F, the distributions of the HOMO and LUMO orbitals of IDTI were similar, as FMO were mainly localized around the IDTI back bone. After the binding of F^−^, the HOMO and LUMO orbital distributions were quite different. Most of the electron wavefunctions became delocalized along the conjugation backbone for the LUMO, which is similar to electron distribution of IDTI. However, the electron distribution of the HOMO of IDTI-2F^−^ was mainly located at both ends of IDTI, particularly the indole units. This result indicates that after the fluoride bonding, upon the excitation of IDTI-2F^−^, an electron transfer from the end indole groups to the conjugated backbone proceeded. The fluoride-bonded indole units seem to exhibit stronger electron-withdrawing properties than indole, which results in a strong push–pull system (core of IDTI-fluoride-bonded indole). Normally, a strong push–pull system could result in a bathochromic shift; however, in this work, a blue shift occurred (Trilling et al., 2019; Zhang et al., 2020).

## Conclusion

In this study, a new type of colorimetric chemosensor dye based on IDTI with high sensitivity toward fluoride anions was designed and investigated. IDTI can interact with fluoride anions to exhibit color changes that are visible to the naked eye; such color changes were from red to orange or yellow under ambient light and from purple to green or blue under 365-nm UV light. The color change could be ascribed to the intermolecular proton transfer between IDTI and F^−^. The spectroscopy studies indicate that IDTI could be used to quantitatively analyze the fluoride concentration with a detection limit of as low as 1 × 10^−7^ M. This work has also demonstrated that IDTI is a promising dye for fluoride chemosensors with the advantages of high sensitivity and naked-eye detection.

## Data Availability Statement

All datasets presented in this study are included in the article/supplementary material.

## Author Contributions

XY wrote the manuscript. XS prepared the materials and carried out in experiments. CW revised the manuscript and reference. YD and PJ helped for the characterization. XJ and YS helped in testing and analysis. XH and LL gave the ideas and supervised the whole work. All authors contributed to the article and approved the submitted version.

## Conflict of Interest

The authors declare that the research was conducted in the absence of any commercial or financial relationships that could be construed as a potential conflict of interest.
